# Comorbidity of Physical and Anxiety Symptoms in Adolescent: Functional Impairment, Self-Rated Health and Subjective Well-Being

**DOI:** 10.3390/ijerph15081698

**Published:** 2018-08-09

**Authors:** Judit Balázs, Mónika Miklósi, Agnes Keresztény, Christina W. Hoven, Vladimir Carli, Camilla Wasserman, Gergö Hadlaczky, Alan Apter, Julio Bobes, Romuald Brunner, Paul Corcoran, Doina Cosman, Christian Haring, Jean-Pierre Kahn, Vita Postuvan, Michael Kaess, Airi Varnik, Marco Sarchiapone, Danuta Wasserman

**Affiliations:** 1 Vadaskert Child and Adolescent Psychiatric Hospital, 1021 Budapest, Hungary; 2 Institute of Psychology, Eötvös Loránd University, 1064 Budapest, Hungary; miklosi.monika@ppk.elte.hu (M.M.); kereszteny.agnes@ppk.elte.hu (A.K.); 3 Heim Pál National Pediatric Institute, 1131 Budapest, Hungary; 4 School of Ph.D. Studies, Semmelweis University, 1085 Budapest, Hungary; 5 Department of Child and Adolescent Psychiatry, New York State Psychiatric Institute, Columbia University, New York, NY 10032, USA; hoven@childpsych.columbia.edu (C.W.H.); camillawasserman@gmail.com (C.W.); 6 Department of Epidemiology, Mailman School of Public Health, Columbia University, New York, NY 10032, USA; 7 National Centre for Suicide Research and Prevention of Mental Ill-Health (NASP), Karolinska Institute, SE-171 77 Stockholm, Sweden; vladimir.carli@ki.se (V.C.); Gergo.Hadlaczki@ki.se (G.H.); Danuta.Wasserman@ki.se (D.W.); 8 Department of Health Sciences, University of Molise, 86100 Campobasso, Italy; marco.sarchiapone@me.com; 9 Feinberg Child Study Center, Schneider Children’s Medical Center, Tel Aviv University, Tel Aviv 6997801, Israel; asapter@gmail.com; 10 Department of Psychiatry, School of Medicine, University of Oviedo; Centro de InvestigaciónBiomédica en Red de Salud Mental, CIBERSAM, 33006 Oviedo, Spain; bobes@uniovi.es; 11 Section Disorders of Personality Development, Clinic of Child and Adolescent Psychiatry, University of Heidelberg, 69117 Heidelberg, Germany; romuald.brunner@med.uni-heidelberg.de (R.B.); michael.kaess@upd.ch (M.K.); 12 Department of Child and Adolescent Psychiatry, University of Regensburg, 93053 Regensburg, Germany; 13 National Suicide Research Foundation, Cork, Ireland; pcorcoran@ucc.ie; 14 Clinical Psychology Department, Iuliu Hatieganu University of Medicine and Pharmacy, 400012 Cluj-Napoca, Romania; doina_octaviancosman@yahoo.com; 15 Research Division for Mental Health, University for Medical Information Technology (UMIT), 6060 Hall in Tirol, Austria; christian.haring@tilak.at; 16 Department of Psychiatry, Centre Hospitalo-Universitaire (CHU) de NANCY, Université H. Poincaré, 54003 Nancy, France; jp.kahn@chu-nancy.fr; 17 Mental Health Department, PINT, University of Primorska, 6000 Koper, Slovenia; vita.postuvan@upr.si; 18 University Hospital of Child and Adolescent Psychiatry and Psychotherapy, University of Bern, 3012 Bern, Switzerland; 19 Estonian-Swedish Mental Health & Suicidology Institute, Ctr. Behav & Hlth Sci, Tallinn University, 10120 Tallinn, Estonia; airiv@online.ee

**Keywords:** anxiety, physical morbidity, self-rated heath, comorbidity, categorical diagnostic model, dimensional diagnostic model, adolescent, SEYLE

## Abstract

Physical disorders and anxiety are frequently comorbid. This study investigates the characteristics of physical disorders, self-rated heath, subjective well-being and anxiety in adolescents. Data were drawn from the *Saving and Empowering Young Lives in Europe* cohort study. From 11 countries 11,230 adolescents, aged 14–16 years were included. Zung Self-Rating Anxiety Scale (SAS), WHO-5 Well-Being Index and five questions prepared for this study to evaluate physical illnesses and self-rated heath were administered. Anxiety levels were significantly higher in adolescents who reported having physical disability (*p* < 0.001, Cohen’s *d* = 0.40), suffering from chronic illnesses (*p* < 0.001, Cohen’s *d* = 0.40), impairments associated to health conditions (*p* < 0.001, Cohen’s *d* = 0.61), or reported poor to very poor self-rated health (*p* < 0.001, Cohen’s *d* = 1.11). Mediational analyses revealed no direct effect of having a chronic illness/physical disability on subjective well-being, but the indirect effects through higher levels of anxiety were significant. Functional impairment related to health conditions was both directly and indirectly (through higher levels of anxiety) associated with lower well-being. The co-occurrence of anxiety and physical disorders may confer a greater level of disability and lower levels of subjective well-being. Clinicians have to screen anxiety, even in a subthreshold level in patients with choric physical illness or with medically unexplained physical symptoms.

## 1. Introduction

Several studies have suggested that the co-occurrence of chronic physical disorders and anxiety is high among children and adolescents [1,2,3,4,5,6,7]. The lifetime prevalence of anxiety disorders among children/adolescents with chronic physical illnesses can be as high as 40% [5,6,8], which is higher than in the general adult population, where it is roughly 28–30% [8,9]. Additionally, adults with anxiety disorders were also found to have higher prevalence of chronic physical illnesses compared to those without anxiety disorders [2]. Moreover medically unexplained physical symptoms were reported in patients with anxiety significantly more often in a community sample [1]. Sareen et al. [7] highlighted that anxiety disorders are positively associated with physical disorders even after adjusting for mood disorders, substance-use disorders and sociodemographic factors. Csupak et al. [10] found in a community sample of adults that generalized anxiety disorder is prevalent among chronic pain conditions, (i.e., arthritis, back pain, and migraines) and comorbidity is associated with greater pain severity.

The most common chronic physical illnesses in children/adolescents are asthma, diabetes, epilepsy, aches and burns and the most prevalent medically unexplained physical symptoms are related to general pains, including headache, stomachache [5,11]. Research shows, that several anxiety disorders are highly present in children and adolescents with physical illnesses [5]. Jones et al. [12] found in a longitudinal study of more than 5000 youth (baseline: in grade 5, follow-up: in grades 7, and 10). At the baseline 28.5% of the youth had experienced a chronic physical health condition and having any chronic physical health condition was related to elevated depressive symptoms in grades 7 and anxiety symptoms in grades 10. After adjusting for previous mental health symptoms, having any condition still predicted anxiety in grades 10.

The interaction between anxiety and physical illnesses can be complex, both causal (bidirectional) and/or synergic (additive). Anxiety disorders can be: (a) psychological consequences of being ill and/or having continuous treatment, (b) direct metabolic/endocrine result of the specific physical illness, (c) present independently as the result of genetic factors, (d) present independently as the result of psychosocial factors (e) a special combination of the factors listed from (a)–(d) sub-points [13,14]. Anxiety can worsen physical illness directly by decreasing immune function and/or with an indirect mechanism by decreasing patient compliance [5,13,14].

Research suggest, that children with chronic physical illnesses or with medically unexplained physical symptoms and with comorbid anxiety tend to report poorer treatment outcome compared to those without anxiety, mainly because anxiety disorders often remain unrecognized [15,16].

In the past decade the concept of self-rated health has gained growing interest, which covers physical, mental and social components [17,18,19,20]. Previous research shows that self-rated heath complaints (i.e., headache, backache, sleeping difficulties, feeling low, perceived stress, etc.)—which refer to symptoms experienced by the individual with or without a defined diagnosis—are common among adolescents and increase with age, especially among girls [21,22,23,24,25,26]. It is interesting, that self-rating health seems to be only modestly correlated with clinical assessments of medical status [27].

As an important measure of individuals’ general state, the concept of quality of life (QoL) and its relationship with physical and mental health disorders has received increased attention over recent years [28,29]. Research shows, that subjective well-being is an important dimension of overall perceived QoL [30]. The definition of well-being refers to both positive psychological experience and optimal functioning [31].

Studies have also suggested that increasing number of the anxiety symptoms is associated with the severity of medically unexplained physical symptoms [32]. An expanding body of literature supports the importance of reconceptualization of the current classification systems—such as International Classification of Mental and Behavioral Disorders (ICD-10) [19] and the Diagnostic and Statistical Manual of Mental Disorders Fifth Edition (DSM-5) [33]—due to their categorical construction and suggests to include dimensional approach into the classification [34,35,36,37]. Subthreshold disorders—symptomatology that fall short of the criteria for a diagnosis according to the classification systems—could function as a hybrid of categorical and dimensional approaches [38,39].

In the *Saving and Empowering Young Lives in Europe* (SEYLE) study we found on a random community sample of adolescents aged 14–16, that 5.8% of them was anxious and additional one-third of them had current subthreshold anxiety, based on screening tools [34,40]. Subthreshold anxiety was associated with an increased burden of disease and suicide risk [34].

Based on the SEYLE study, using both categorical and dimensional diagnostic models of anxiety, the aims of the current study were to explore in European adolescents the impact of comorbid anxiety and physical health on self-rated health and subjective well-being. We assumed that adolescents with unfavorable health conditions (having a chronic illness, having a physical disability, and reporting that these conditions hinder daily activities) have higher levels of anxiety and lower levels of subjective well-being. Furthermore, we hypothesized that anxiety mediates the relationships between health-conditions and subjective well-being.

## 2. Materials and Methods

### 2.1. Participants

The SEYLE study is a randomized controlled trial (RCT), which has been registered at the German Clinical Trials Register (DRKS00000214) [40]. The detailed methodology including sampling procedures and measures of the SEYLE study were previously described [40]. SEYLE’s sample of adolescents (aged 14–16 years) is from 11 European countries: Austria, Estonia, France, Germany, Hungary, Ireland, Israel, Italy, Romania, Slovenia and Spain, with Sweden serving as the coordinating center. Ethical approval was obtained from each site’s local ethics committee. Local school authorities granted access to randomly selected school (s) and informed assent and consent were obtained, as required.

In this study we analyzed data from 11,230 adolescents (57% girls), mean age was 14.89 years (*SD* = 0.87). Characteristics of the sample regarding country of pupils are shown in Table 1.

### 2.2. Data Collection

A self-report questionnaire was administrated, which included well-established measures and items developed for SEYLE [40]. Symptoms of current anxiety were assessed by Zung Self-Rating Anxiety Scale (SAS) [41]. Ratings for the 20 items of the SAS (rated 0 to 4) were summed together. Adolescents were also divided into three groups based on the transformed [42] SAS Index Score: ≥60 = anxious; 45–60 = subthreshold-anxious; <45 = non-anxious.

Subjective well-being was assessed by the WHO-5 Well-Being Index, a short, self-administered questionnaire covering five positively worded items, related to positive mood, vitality, and general interests [43]. Each of the five items is rated on a 6-point Likert scale from 0 (=at no time present) to 5 (=all of the time present).

To evaluate physical illnesses and self-rated health the following five questions were included into the questionnaire: “(1) Have you seen a doctor in the last 12 months? (2) Do you have a physical disability? (3) Do you suffer from any chronic illness? (4) Do these discomforts lower your ability or hinder other daily activities? (5) Overall, how would you describe your state of health these days?” The first three questions had to answer with “Yes” or “No”, while the possible answers to “question 4” are “Yes, certainly”/“Yes, somewhat”/“Not at all” and to “question 5” are “Very good”/“Good”/“Fair”/“Poor”/“Very poor”.

### 2.3. Analyses

Frequencies of pupils showing marked/severe and subthreshold anxiety symptoms, as well as mean SAS scores are reported by groups according to YES/NO answers to the health-related questions. For group comparisons, chi-square tests were used for categorical variables and independent sample *t*-tests for continuous variables.

Furthermore, we tested mediational models in which anxiety mediate the relationships between health-conditions (having a chronic illness, having a physical disability, and reporting that these conditions hinder daily activities) and subjective well-being. We calculated direct and indirect effects using the approach provided by Hayes [44].

## 3. Results

Among all 11,230 adolescents, 9639 (85.8%) saw a doctor in the last 12 months, 317 (2.8%) described their state of health poor or very poor, 315 (2.8%) reported of having physical disability and 1684 (15.0%) suffered from chronic illness. Among the pupils who suffered from chronic illness 849 (50.4%) reported that these discomforts lower their ability somewhat or certainly.

### 3.1. Prevalence of Subthreshold and Full Anxiety in the Study Groups

Table 2 shows prevalence of full and subthreshold anxiety in the groups. There was no significant difference in the prevalence of anxiety between adolescents who saw and who did not see a doctor in the past 12 months. The difference in the prevalence of subthreshold anxiety in the two groups was significant, but effect size was small. Among those who described their state of health poor or very poor, the both prevalence of anxiety and subthreshold anxiety were significantly higher than in adolescents reporting good or very good state of health. Effect sizes were small. Adolescents with and without a physical disability differed significantly in the prevalence of anxiety and subthreshold anxiety, however the effect sizes were small. Among adolescents who suffered from a chronic illness, the prevalence of anxiety and subthreshold anxiety were higher than among those who reported of not having a chronic health condition, however the effect sizes were small. Finally, there were significant differences of small effect size in the prevalence of anxiety and subthreshold anxiety among adolescents who reported that these discomforts lower their ability somewhat or certainly, and those who reported that these discomforts did not lower their ability (Table 2).

### 3.2. Differences in Anxiety and Subjective Well-Being in the Study Groups

There were no significant differences in SAS and WHO-5 Well-Being Index scores among those who saw or did not see a doctor in the last 12 months. Among those who described their state of health poor or very poor, SAS scores were significantly higher and WHO-5 Well-Being Index scores were significantly lower than among those who described their state of health fair, good or very good. Effect sizes were large. Adolescents who reported of having a physical disability scored significantly higher on SAS and scored significantly lower on WHO-5 Well-Being Index than those who reported of not having a physical disability. Similarly, adolescents who suffered from a chronic illness, showed significantly higher mean SAS score and lower mean WHO-5 Well-Being Index score than adolescents who did not suffer from a chronic illness. Finally, among those who reported that these discomforts lower their ability somewhat or certainly, SAS scores were significantly higher and WHO-5 Well-Being Index scores were significantly lower than among those who reported that these discomforts did not lower their everyday abilities. Effect sizes were small to medium (Table 3).

### 3.3. Anxiety as a Mediator between Health Conditions and Well-Being

**Figure 1:**
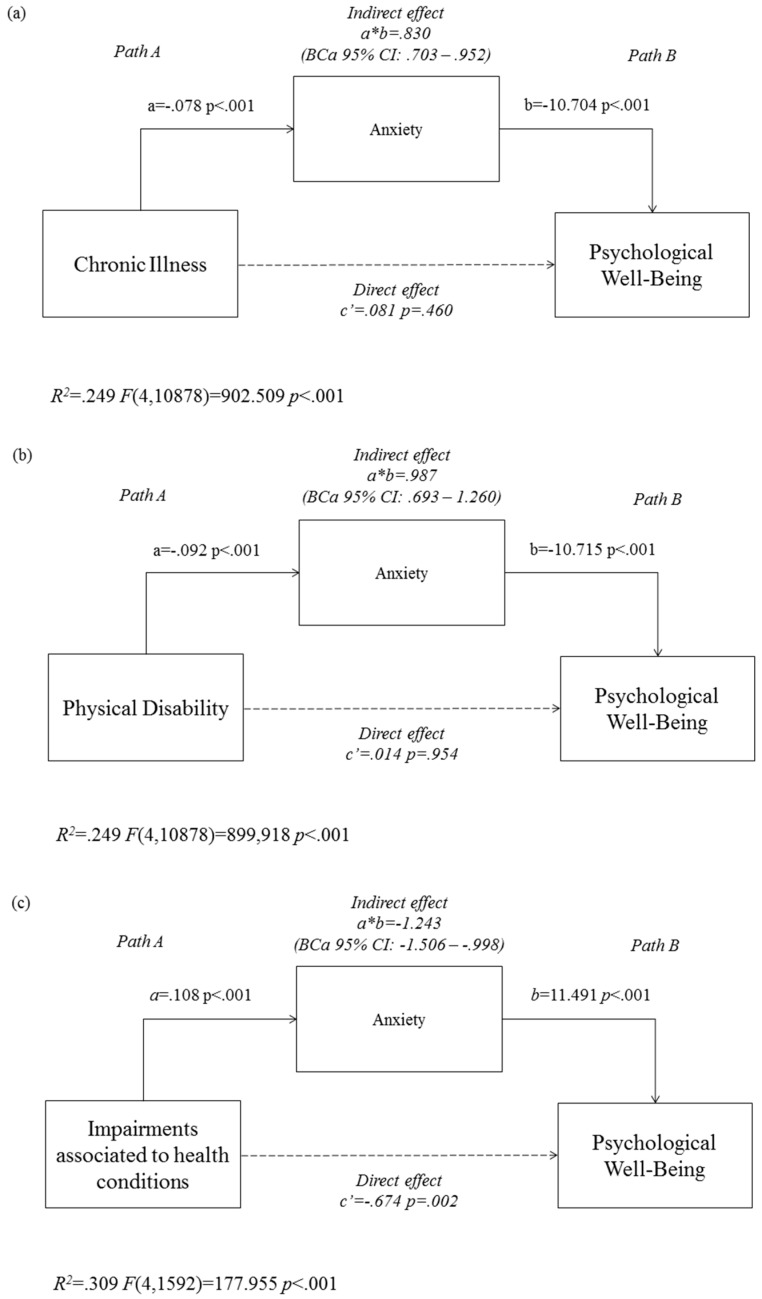
Indirect effects of having a chronic illness (**a**), having a physical disability (**b**), as well as having impairments associated with these health conditions (**c**), on subjective well-being (WBI-5 scores), as mediated by anxiety (SAS scores) (unstandardized regression coefficients). *Note.* SAS: Zung Self Rating Anxiety Scale. WBI-5: WHO-5 Well-being Index. *BCa 95% CI*: bias corrected and accelerated 95% Confidence Interval. Number of Bootstrap Resample: 1000.

Results of the mediation analyses are presented in Figure 1a–c. We calculated direct and indirect effects to test the hypothesis that the level of anxiety mediate the relationship between health conditions and subjective well-being. The direct effect of having a chronic illness on well-being was not significant (*c*’ = 0.081, *p* = 0.460). On the other hand, having a chronic illness was related to increased levels of anxiety, which in turn was associated with lower levels of well-being (Figure 1a), and the indirect effect of having a chronic illness on subjective well-being through anxiety was significant (a*b = 0.830, *BCa 95% CI*: 0.703–0.952).

Similarly, we found no direct effect of having a physical disability on subjective well-being (*c*’ = 0.014, *p* = 0.954), but there was a significant indirect effect through anxiety (a*b = 0.987, *BCa 95% CI*: 0.693–1.260). Paths A and B in Figure 1b indicate that having a physical disability was related to higher levels of anxiety, which in turn was associated with lower levels of well-being.

Among those, who reported having a chronic illness, we tested a third mediational model whether anxiety level mediates the relationship between impairment associated to health conditions and subjective well-being. Figure 1c presents, that both the direct (*c*’ = −0.674, *p* = 0.002) and the indirect effect through anxiety (a*b = 1.243, *BCa 95% CI*: −1.506–−0.998) were significant; adolescents reporting that health conditions lower their ability somewhat or certainly showed increased levels of anxiety, which in turn was related to lower subjective well-being.

## 4. Discussion

Our study found in a large random sample, that as many as 15% of European adolescents suffer from a chronic illness and half of them report that these discomforts lower their ability to perform daily tasks. These results have public health importance in drawing the attention of clinicians and decision makers to put focus on adolescents with chronic illnesses.

The focus of our research was on examining the impact of comorbid physical and anxiety symptoms in adolescents. First, we categorized the level of anxiety based on the SAS score as non-anxious, subthreshold-anxious and anxious. There were significant differences in the prevalence of anxiety with small effect size between those who: (a) suffered or didn’t suffer from chronic illness, (b) described their state of health poor/very poor and good/very good, and (c) reported that the discomforts due to their chronic illnesses lower or do not lower their daily abilities somewhat/certainly. Additionally, we found that there were significant differences already in the level of subthreshold anxiety with small effect size between those who: (a) suffered or didn’t suffer from chronic illness, (b) described their state of health poor/very poor and good/very good, and (c) reported that the discomforts due to their chronic illnesses lower or do not lower their daily abilities somewhat/certainly. All these data show that in the case of physical illnesses and poor self-rated health physicians have to screen the symptoms of anxiety already in a subthreshold level. The data in this paper supports previous studies, which underline the role of comorbidity of anxiety with psychical symptoms in adolescents [1,2,3,4,5,6,7]. Moreover, the current study highlights the importance of recognition and appropriate intervention already for subthreshold anxiety in adolescents with physical disorders and/or with perceived discomfort. Knowing from previous research, that subthreshold disorders can increase the probability of full disorders, based on the current results the recognition of subthreshold anxiety in this special paediatric population seems to be core mental health issues [39].

Second, we used dimensional diagnostic model based on the SAS. The level of anxiety was significantly higher with small effect size among those who: (a) reported having physical disability compared to those who reported of not having physical disability; (b) suffered from chronic illness, compared to those who did not suffer from chronic illness. Additionally, using dimensional diagnostic model our data show that the level of anxiety was significantly higher with medium effect size among those who reported that the discomforts due to their chronic illnesses lower their daily ability somewhat or certainly compared to those who reported that the discomforts due to their chronic illnesses did not lower their ability. Moreover, the level of anxiety was significantly higher with high effect size among those who described their state of health poor or very poor compared to those who described their state of health fair, good or very good. This study shows that functional impairment and self-rated health has the strongest association with anxiety in young people with physical symptoms. Considering previous research suggesting that children with physical and comorbid, mainly unrecognized anxiety have pooper treatment outcome [16], this information can be essential.

Important results of the current research are that those adolescents reported significantly lower subjective well-being who: (a) suffered from chronic illness, compared to those ones, who did not, (b) described their state of health poor/very poor, compared to those ones, who described it as good/very good, (c) reported that the discomforts due to their chronic illnesses lower their daily abilities somewhat/certainly, compared to those ones, who reported that the discomforts did not lower their daily abilities. While subjective well-being is considered as an important aspect of the quality of life, these data confirm, that health conditions have a major impact on European adolescents.

Furthermore we examined in a mediation analyses whether there is a direct effect of health conditions on subjective well-being and we analysed, if the level of anxiety—which as we described above was found high in this population, similarly to previous research (i.e., [3,4,5,6])—mediates the relationship between health conditions and subjective well-being. Interestingly we did not find a direct effect of having a chronic illness or a physical disability on well-being, but we did between self-reported impairment associated to health conditions and well-being. These results suggest the need for the implementation of routine measuring of functional impairment in adolescent with chronic illnesses or with physical disability.

Additionally, both of having a chronic illness or a physical disability was related to a significantly higher level of anxiety, which was associated with significantly lower level of well-being. Furthermore, we found that the level of anxiety mediated the relationship between self-reported impairment associated to health conditions and subjective well-being as well. Based on these results anxiety seems to have a key role in lower well-being of adolescents with chronic illnesses and physical disabilities. Consultation liaison should have a special focus on it. Moreover, comprehensive evidence-based methods are needed for assessing anxiety in pediatric chronic physical disorders [45]. All these should be considered in further prevention and intervention approaches.

Neither using categorical, nor using dimensional diagnostic model we did not found differences in the level of anxiety between those who saw or did not see a doctor in the last 12 months. Furthermore, we did not find differences in the mean well-being score between those who saw or did not see a doctor in the last 12 months. The explanation could be, that the reason of seeing a doctor, especially at this age, can include with a high prevalence less severe cases, like smaller infections, when the child cannot attend the school and needs a certificate to the school from a doctor. Based on our data just measuring the number of medical visits in this age group is not enough to plan any prevention programs. 

Regarding to the on-going debate, whether categorical or dimensional classification systems should be developed, the results of the current study on young people with physical symptoms support that dimensional approach seems to be a more sensitive method of evaluating psychopathology. However, our data suggests as several previous research recently did, that subthreshold psychopathology, which is a categorical approach again, could serve as a bridge between categorical and dimensional classification of mental disorders [38,39]. At this point it is important to remember that anxiety exists on a continuum from normal to pathological [32] and when the symptoms of anxiety increase to such severity that the individual can no longer function effectively in everyday life, the syndrome should be considered to be an anxiety disorder [9,33]. The typical development of adolescents can include a higher, but not clinical level of anxiety, as in this life period there are several “normal” stress factors, for example becoming less dependent from the parents, finding their role among peers, making the decision, what profession to choose, which increase levels of anxiety.

The limitations of the study require consideration. At first the cross-sectional design has its given limitations. Since cross-sectional data represent a “snap-shot in time”, our results cannot indicate (one-way directional) causality or symptom development over time. Accordingly, the present results do not explain whether perceived stress caused health complaints, or vice versa. Additionally, the measures were self-report based, including the disability outcome measure. Future studies would benefit from using clinician-administered assessments in longitudinal study designs, including the measure of disability or other objective evidence such as breaks and returns to school work.

## 5. Conclusions

To conclude in line with the WHO definition of health as the combination of physical, mental and social factors [46], based on our data we would like to highlight that the comorbidity of physical and anxiety symptoms is high. Knowing the high comorbidity of chronic physical and anxiety symptoms in childhood and the comorbid presence of these symptoms could be associated with concurrent and long-term impairment [45], the topic of this papers is a critically important health problem. Therefore, the practical implications of the current study are that clinicians routinely and repeatedly have to screen, assess and properly treat the symptoms of anxiety in patients with chronic physical illness. The clinicians’ focus on both conditions could be important to develop appropriate, targeted interventions and may increase the likelihood of improved outcomes. Based on our findings we emphasize that adolescents with physical and psychiatric symptoms—even on the subthreshold level—require evaluation of functional impairment, self-rated health and subjective well-being as it is essential for secondary prevention.

## Figures and Tables

**Table 1:** Sample characteristics regarding country of pupils.

Country of Pupils	*N*	%
Austria	916	8.2
Estonia	1004	8.9
France	966	8.6
Germany	1395	12.4
Hungary	953	8.5
Ireland	879	7.8
Israel	818	7.3
Italy	1122	10.0
Romania	1036	9.2
Slovenia	1133	10.1
Spain	1008	9.0

**Table 2:** Prevalence of anxiety in the study groups.

Health Condition	*Total N (%)*	Levels of Anxiety According to SAS			
		*Anxious N (%)*	*χ²; p; ϕ*	*Subthreshold Anxious N (%)*	*χ²; p; ϕ*
Saw a doctor in the last 12 months	9639 (85.8)	461 (4.8)	χ² < 0.001; *p* = 0.995	2361 (24.5)	χ² = 4.967; *p* = 0.026; ϕ = 0.022
Did not see a doctor in the last 12 months	1591 (14.2)	76 (4.8)		349 (21.9)	
State of health poor or very poor	317 (2.8)	98 (30.9)	χ² = 489.282; *p* < 0.001; ϕ = 0.209	126 (39.7%)	χ² = 122.448; *p* < 0.001; ϕ = 0.107
State of health fair, good or very good	10,913 (97.2)	439 (4.0)		2584 (23.7)	
Physical disability	315 (2.8)	36 (11.4)	χ² = 31.446; *p* < 0.00; ϕ = 0.053	102 (32.4)	χ² = 19.045; *p* < 0.001; ϕ = 0.042
No physical disability	10,915 (97.2)	501 (4.6)		608 (23.2)	
Chronic illness	1684 (15.0)	162 (9.6)	χ² = 101.845; *p* < 0.001; ϕ = 0.095	532 (31.6)	χ² = 86.624; *p* < 0.001; ϕ = 0.090
No chronic illness	9546 (58.0)	375 (3.9)		2178 (22.8),	
Chronic illness lowering ability	849 (50.4)	122 (14.4)	χ² = 44.664; *p* < 0.001; ϕ = 0.164	326 (38.4)	χ² = 62.697; *p* < 0.001; ϕ = 0.204
Chronic illness does not lowering ability	835 (49.6)	38 (4.7)		196 (24.2)	

Notes: *N* = 11,230. SAS: Zung Self Rating Anxiety Scale.

**Table 3:** Differences in anxiety and subjective well-being in the study groups.

Health Condition	SAS		WBI-5	
	*Mean (SD)*	*t; p; Cohen’s d*	*Mean (SD)*	*t ; p; Cohen’s d*
Saw a doctor in the last 12 months	41.09 (9.23)	*t* = 1.646; *p* = 0.10	15.72 (4.69)	*t* = 0.043; *p* = 0.963
Did not see a doctor in the last 12 months	40.68 (9.18)		15.73 (5.24)	
State of health poor or very poor	52.61 (12.39)	*t* = 17.00; *p* < 0.001; *d* = 1.11	10.99 (5.42),	*t* = 15.768; *p* < 0.001; *d* = 0.95
State of health fair, good or very good	40.70 (8.89)		15.86 (4.68)	
Physical disability	44.65 (10.95)	*t* = 5.98; *p* < 0.001; *d* = 0.40	14.64 (5.50),	*t* = 3.518; *p* < 0.001; *d* = 0.22
No physical disability	40.93 (9.14)		15.75 (4.74)	
Chronic illness	44.10 (10.77)	*t* = 13.001; *p* < 0.001; *d* = 0.40	14.87 (5.16)	*t* = 7.441; *p* < 0.001; *d* = 0.22
No chronic illness	40.49 (8.81)		15.87 (4.68)	
Chronic illness lowering ability	47.11 (11.39)	*t* = 12.452; *p* < 0.001; *d* = 0.61	13.77 (5.15)	*t* = 9.34; *p* < 0.001; *d* = 0.46
Chronic illness does not lowering ability	40.86 (8.98)		16.07 (4.87)	

Notes: *N* = 11,230. SAS: Zung Self Rating Anxiety Scale. WBI-5: WHO-5 Well-being Index.
